# The proto-oncogene Mer tyrosine kinase is a novel therapeutic target in mantle cell lymphoma

**DOI:** 10.1186/s13045-018-0584-6

**Published:** 2018-03-20

**Authors:** Cunzhen Shi, Xiangqun Li, Xiaogan Wang, Ning Ding, Lingyan Ping, Yunfei Shi, Lan Mi, Yumei Lai, Yuqin Song, Jun Zhu

**Affiliations:** 1 Key laboratory of Carcinogenesis and Translational Research (Ministry of Education), Department of Lymphoma, Peking University Cancer Hospital & Institute, No. 52 Fucheng Road, Haidian District, Beijing, 100142 China; 2 Beijing Doing Biomedical Technology Co., Ltd, Songyubei Road, Chaoyang District, Beijing, 100101 China; 3 Key Laboratory of Carcinogenesis and Translational Research (Ministry of Education), Department of Pathology, Peking University Cancer Hospital & Institute, No. 52 Fucheng Road, Haidian District, Beijing, 100142 China; 4 Key Laboratory of Carcinogenesis and Translational Research (Ministry of Education), Peking University Cancer Hospital & Institute, No. 52 Fucheng Road, Haidian District, Beijing, 100142 China

**Keywords:** Mer tyrosine kinase, Mantle cell lymphoma, Therapeutic target, UNC2250

## Abstract

**Background:**

Mantle cell lymphoma (MCL) is an incurable B cell-derived malignant tumor with a median overall survival of 4–5 years. Mer tyrosine kinase (MerTK) has been reported to be aberrantly expressed in leukemia, melanoma, and gastric cancer, and plays a pivotal role in the process of oncogenesis. This study assessed the role of MerTK in MCL for the first time.

**Methods:**

Immunohistochemistry and western blot were performed to figure out expression of MerTK in MCL. MerTK inhibition by either shRNA or treatment with UNC2250 (a MerTK-selective small molecular inhibitor) was conducted in MCL cell lines. MCL-cell-derived xenograft models were established to evaluate the anti-tumor effects of UNC2250 in vivo.

**Results:**

MerTK was ectopically expressed in four of six MCL cell lines. Sixty-five of 132 (48.9%) MCL patients showed positive expression of MerTK. MerTK inhibition by either shRNA or treatment with UNC2250 decreased activation of downstream AKT and p38, inhibited proliferation and invasion in MCL cells, and sensitized MCL cells to treatment with vincristine in vitro and doxorubicin in vitro and in vivo. UNC2250 induced G2/M phase arrest and prompted apoptosis in MCL cells, accompanied by increased expression of Bax, cleaved caspase 3 and poly (ADP-ribose) polymerase, and decreased expression of Bcl-2, Mcl-1 and Bcl-xL. Moreover, UNC2250 delayed disease progression in MCL-cell-derived xenograft models.

**Conclusions:**

Our data prove that ectopic MerTK may be a novel therapeutic target in MCL, and further pre-clinical or even clinical studies of UNC2250 or new MerTK inhibitors are essential and of great significance.

**Electronic supplementary material:**

The online version of this article (10.1186/s13045-018-0584-6) contains supplementary material, which is available to authorized users.

## Background

Mantle cell lymphoma (MCL), an aggressive B cell-derived malignant tumor of the hematological and lymphatic systems, accounts for 6–8% of non-Hodgkin lymphoma [1] and is associated with poor prognosis with a median overall survival (OS) of 4–5 years [2]. Most patients with MCL experience a very short regression duration after standard first-line therapy. In spite of various salvage therapies for MCL, such as temsirolimus, bortezomib, ibrutinib, lenalidomide, and autologous hematopoietic stem cell transplantation (ASCT) [3–9], there is still no curative treatment available for MCL. Therefore, it is important to exploit new therapeutic targets or regimens to further improve the prognosis of MCL.

Mer tyrosine kinase (MerTK), also known as RP38, c-Eyk, c-mer, and Tyro12, was first cloned from a human B lymphoblastoid expression library by Graham et al. [10] and is one of the TAM (Tyro-3, Axl, and MerTK) receptor tyrosine kinase (RTK) family [11]. As in other RTK family proteins, aberrant expression of MerTK in various malignant tumors, such as melanoma [12, 13], gastric cancer [14], leukemia [15–17], and lung cancer [18], plays a pivotal role in the process of oncogenesis. By binding to its corresponding ligand (Gas6, protein S, tubby, tubby-like protein 1, and galectin-3), auto-phosphorylation sites (Y749, Y753, and Y754) of MerTK are activated and lead to activation of downstream signaling pathways including extracellular signal-regulated kinase (ERK)1/2, AKT, p38, focal adhesion kinase (FAK) and signal transducer and activator of transcription (STAT)6 [11]. MerTK is not expressed in normal B or T lymphocytes, but in neoplastic B or T lymphocytes [19]. Within the hematopoietic malignant tumors, ectopic expression of MerTK has been reported in pre-B cell acute lymphoblastic leukemia (B-ALL), T cell acute lymphoblastic leukemia (T-ALL), and acute myeloid leukemia (AML) [15–17]. shRNA-mediated MerTK knockdown or a series of MerTK-selective small molecular inhibitors, such as UNC1062, UNC569, and UNC2025, reduces activation of downstream signaling, inhibits proliferation and invasion, and promotes apoptosis in tumor cells [12, 14, 20–23]. Therefore, ectopic MerTK may be a pivotal promoter in the development of B or T cell-derived hematopoietic malignant tumors.

Differential gene expression analysis between MCL cells and normal B cell populations identified MerTK as an upregulated oncogene in MCL [24], but there have been no further studies about the function of MerTK in MCL. Our data revealed that MerTK was ectopically expressed in MCL patients and MCL cell lines. MerTK inhibition by either shRNA or treatment with UNC2250, a MerTK-selective small molecular tyrosine kinase inhibitor, suppressed pro-survival signaling, proliferation, invasion, and migration in MCL cells and promoted chemosensitivity to common chemotherapeutic agents. Additionally, UNC2250 promoted apoptosis and induced G2/M phase arrest in MCL cells and significantly delayed disease progression in MCL-cell-derived xenograft models. These results suggest that ectopic MerTK is a novel therapeutic target in MCL.

## Methods

### Clinical samples and immunohistochemistry (IHC)

Patients’ samples were collected after obtaining informed consent in accordance with the Declaration of Helsinki. All 132 patients were newly diagnosed or had received treatment between January 1, 2001, and June 1, 2017, in the Peking University Cancer Hospital & Institute. Survival analysis was performed on patients treated with R-CHOP-like regimens (R-CHOP, R-CHO, R-CHOPE, and R-mini-CHOP) as first-line therapy and did not undergo ASCT. Overall survival (OS) was defined as the interval between treatment and date of death, and progression-free survival (PFS) was defined as the interval between treatment and disease progression. The detailed clinical characteristics of patients are listed in Additional file 1: Table S1. Immunohistochemistry stains for MerTK were performed as the standard streptavidin-biotin-peroxidase-immunostaining procedure [18]. Primary antibody to MerTK was listed in Additional file 1: Table S2. This study was approved by the Review Board of the Peking University Cancer Hospital & Institute.

### Cell culture and knockdown of MerTK via RNAi

Z-138, Mino, JVM-2, Granta519, JeKo-1, and JVM-13 cell lines were generously provided by Dr. Fu, University of Nebraska Medical Center, USA. All cell lines were cultured in low-glucose Dulbecco’s modified Eagle’s medium (DMEM) (Gibco, Life Technologies, CA, USA) supplemented with 10% fetal bovine serum (FBS) (Gibco, Life Technologies) and penicillin/streptomycin (Gibco) (complete DMEM; cDMEM). Identification of all MCL cell lines was confirmed by short tandem repeat DNA fingerprinting analysis (Applied Biosystems, Foster City, CA, USA). Human peripheral blood mononuclear cells (PBMCs) were freshly isolated from 20 ml blood from healthy volunteers using Lymphoprep (Axis Shield, Oslo, Norway). Normal human B cells were sorted from the PBMCs using a B Cell Isolation Kit II (Miltenyi Biotec, Mecklenburg-Vorpommern, Germany). Lentiviral vectors (GV248) containing green fluorescent protein (GFP) (shControl) or short hairpin RNA (shRNA) sequence targeting MerTK (shMerTK, Oligo ID: TRCN0000000862; shMerTK 4, Oligo ID: TRCN0000000865) were obtained from Genechem (Shanghai, China). Z-138, Mino, and JVM-2 cells were infected with shControl, shMerTK, or shMerTK 4 at MOI 1: 50 and cultured for > 72 h to be used for the downstream experiments.

### Western blot and signaling assays

Cells infected with shControl, shMerTK, or shMerTK 4 were harvested after being cultured for ≥ 72 h. Cells treated with UNC2250 (Selleck, Houston, TX, USA) at indicated concentrations were cultured in cDMEM for 1 h for phosphorylation assays or 24 h for proteins associated with apoptosis and cell cycle. Cell lysates were prepared, and signaling proteins were detected by western blot as previous described [25]. Primary and secondary antibodies were listed in Additional file 1: Table S2. Immunoblot imaging was performed by image processing and analysis software (Fusion SL; Vilber Lourmat Deutschland GmbH, Eberhardzell, Germany).

### Cell proliferation assays

For MerTK knockdown, after being infected with shControl, shMerTK, or shMerTK 4 for > 72 h, cells were plated in triplicate at a density of 2000 cells per 100 μl in 96-well black base microplates and cultured for 0, 24, 48, 72, and 96 h. For sensitivity tests, 2000 cells per 100 μl in 96-well black base microplates were cultured in the absence (vehicle) or presence (dosing) of UNC2250 for 72 h. Viable cells were measured by Cell Titer-Glo Luminescent Cell Viability Assay system (Promega, Madison, WI, USA) according to the manufacture’s protocol, and luminescent signals were measured by LMax II (Molecular Devices, Sunnyvale, CA, USA). Inhibition rates were calculated according to the following formula: inhibition rates = (1 − dosing/vehicle) × 100%.

### Invasion and migration assays

Cells were infected with shControl or shMerTK for > 72 h or treated with vehicle or indicated concentrations of UNC2250 for 2 h in FBS-free DMEM. For invasion assays, cells were seeded into Corning BioCoat Matrigel invasion chambers with an 8.0-μm polyethylene terephthalate membrane (Costar; Corning Incorporated, Corning, NY, USA) in 24-well plates; for migration assays, cells were seeded into Transwell with 8.0 μm pore polycarbonate membrane insert (Costar; Corning Incorporated, Corning, NY, USA). Cells in the upper chamber were cultured in FBS-free DMEM, while 30% FBS was added to the lower chamber. After 24 h, cells invading or migrating into the lower chamber were harvested and resuspended in 100 μl DMEM. Afterwards, cells were plated in 96-well black base microplates, and viable cells were measured as in the cell proliferation assays. Invasive abilities or migration abilities were determined by the number of viable cells invading or migrating into the lower chamber.

### Apoptosis and cell cycle assays

Cells were treated with vehicle or indicated concentrations of UNC2250 for 12, 24, and 48 h for apoptosis assays or for 24 h for cell cycle analysis. For apoptosis assays, cells were stained with annexin-V-FITC and propidium iodide (PI) (Dojindo Laboratories, Kumamoto, Japan) according to the protocol. For cell cycle assays, cells were stained with PI staining buffer (Sigma–Aldrich, Darmstadt, Germany) as previously described [25]. All samples for apoptosis and cell cycle assays were analyzed by BD Accuri C6 flow cytometer (BD Biosciences, San Jose, CA, USA). The results of cell cycle assays were reanalyzed by ModFit LT software (Verity Software House, Topsham, ME, USA).

### MCL-cell-derived xenograft model

Female non-obese diabetic/severe combined immunodeficient (NOD/SCID) mice aged 7–8 weeks were purchased from HFK Bioscience Co. Ltd. (Beijing, China). Animal experiments were conducted in accordance with the Guide for the Care and Use of Laboratory Animals and were approved by the Peking University Cancer Hospital & Institute. Each NOD/SCID mouse was subcutaneously inoculated with 10^7^ Z-138 cells suspended in 0.1 ml PBS on the right side of the back to establish a subcutaneous tumor model. When the tumor volume grew to an average of 100–150 mm^3^, mice were randomly distributed into groups according to tumor size and weight of mice. UNC2250 (25, 50, or 75 mg/kg) or saline (vehicle) was administered once daily at a dose of 10 ml/kg by oral gavage. For combination treatment, UNC2250 50 mg/kg or saline (vehicle) was administered once daily at a dose of 10 ml/kg by oral gavage, vincristine 0.5 mg/kg, or doxorubicin 3 mg/kg or vehicle was administered once daily at a dose of 10 ml/kg by intraperitoneal injection. The weight and tumor size of the mice were measured twice weekly after initiation of treatment. Tumor volume was calculated according to the following formula: *V* = *ab*^2^/2 (*a* and *b* denote respectively long and short diameters of the tumor). Mice were euthanized upon development of advanced tumor (volume > 3000 mm^3^ or average tumor volume of a group of animals > 2000 mm^3^, weight loss > 20%, persistent bleeding, and decreased activity). Tumor tissue samples collected from all groups at 4 h after the last dose were embedded in paraffin for IHC. Phosphorylated MerTK in tumor tissues were detected by IHC.

### Chemosensitivity assays

Cells were plated in triplicate at a density of 2000 cells per 100 μl in 96-well black base microplates. For MerTK knockdown, cells infected with shControl or shMerTK were cultured in the absence (vehicle) or presence (dosing) of vincristine or doxorubicin for 72 h. For UNC2250 inhibition, cells were cultured in cDMEM containing vehicle, or vincristine (doxorubicin), or UNC2250, or combination of vincristine (doxorubicin) and UNC2250 at indicated concentrations for 72 h. Inhibition rates were calculated as in the cell proliferation assays. The combination index values were calculated using CalcuSyn software and were based on that described by Chou and Talalay [26].

### Statistical analysis

All experiments in vitro were repeated at least three times. SPSS Statistics version 20 was used to analyze correlation between clinical parameters and MerTK expression in MCL patients. Otherwise, statistical analyses were performed using GraphPad Prism version 6.01. Data were presented as the mean ± SEM. Data were analyzed using an unpaired *t* test for comparisons of two cohorts. One-way ANOVA was used to analyze the remaining data. *P* < 0.05 was considered to be significant.

## Results

### MerTK was ectopically expressed in MCL cell lines and patients’ samples

**Fig. 1:**
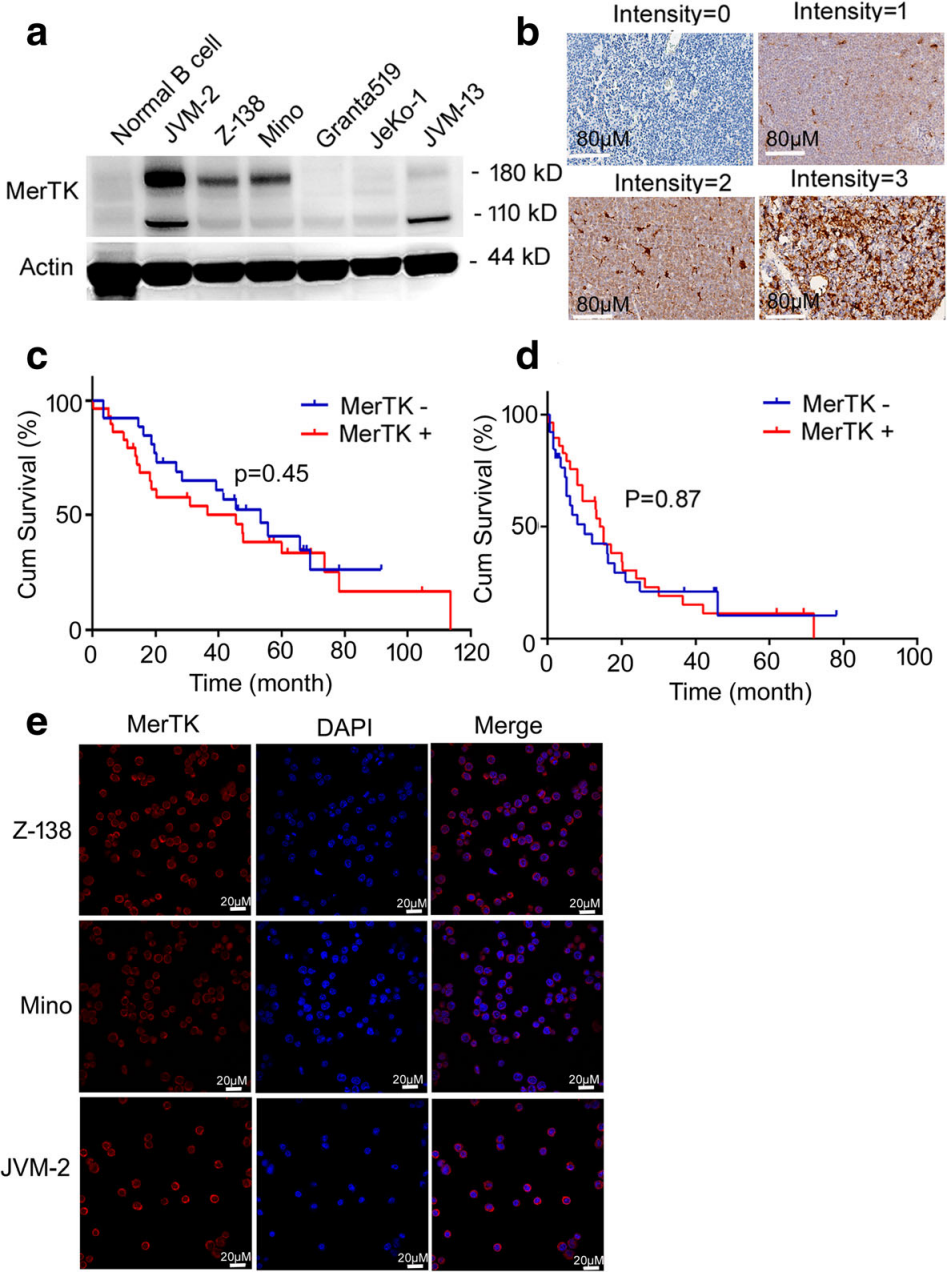
MerTK was ectopically expressed in MCL cell lines and patients’ samples. **a** MerTK expression in MCL cell lines and normal B cells was detected by western blot. Actin is shown as a loading control. JVM-2 and JVM-13 expressed MerTK at bands 180 and 110 kD, whereas Z-138 and Mino cells expressed MerTK at bands 180 kD. **b** Representative pictures of immunohistochemistry staining for MerTK in sections of paraffin-embedded MCL tissues. **c**, **d** Kaplan–Meier curves for OS (**c**) and PFS (**d**) of 55 MCL patients receiving R-CHOP-like regimens according to MerTK expression. **e** MerTK was located in cell membrane in Z-138, Mino, and JVM-2 cells. MerTK expression (red) was detected by immunofluorescence

To figure out expression and function of MerTK in MCL, we analyzed MerTK expression in MCL cell lines by western blot and in samples collected from 132 newly diagnosed or relapsed MCL patients by IHC. Western blot showed that normal B cells, JeKo-1, and Granta519 cells did not express MerTK, while Z-138, Mino, JVM-2, and JVM-13 ectopically expressed MerTK at a medium to high level (Fig. 1a), so Z-138, Mino, and JVM-2 cells were selected for further experiments. Among 132 MCL patients, 65 (48.9%) showed positive expression of MerTK (positive percentage > 10%, Fig. 1b). We analyzed the correlation between MerTK expression and clinical features of 55 patients who received R-CHOP-like regimens as first-line therapy. Respective median OS of the MerTK-negative group or the positive group was 53.2 and 36.5 months (*P* = 0.45) (Fig. 1c), and median PFS was 20.1 and 21.3 months (*P* = 0.87) (Fig. 1d), respectively. These data suggested that MerTK expression had little effect on OS and PFS in this group of patients. MerTK had no correlation with age, sex, complete response (CR), overall response (OR), international prognostic index (IPI), stage, or B symptoms (Additional file 1: Table S1). The confocal immunofluorescence (Additional file 2: Supplementary Methods) results showed that MerTK was located on cell surface of Z-138, Mino, and JVM-2 cells (Fig. 1e).

### MerTK knockdown by shRNA reduced activation of downstream signaling and inhibited proliferation, invasion, and migration in MCL cells

**Fig. 2:**
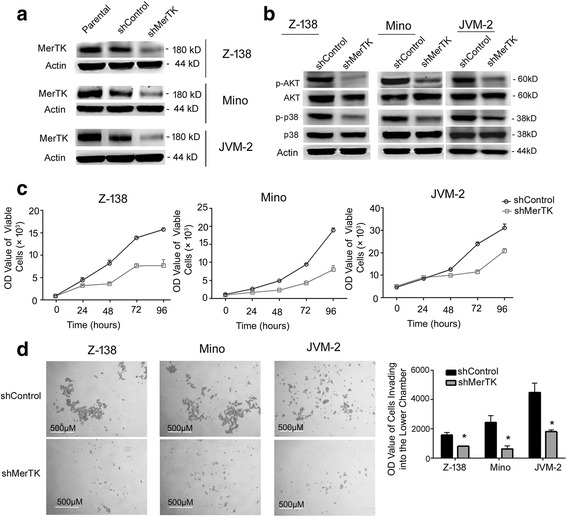
MerTK knockdown mediated by shRNA suppressed downstream signaling pathways, proliferation, and invasion in MCL cells. **a** MerTK knockdown by shRNA was validated by western blot in Z-138, Mino, and JVM-2 cells. Actin is shown as a loading control. **b** Downstream signaling in MerTK knockdown cell lines was evaluated by western blot. Western blot indicated that phosphorylation of AKT (p-AKT) and p38 (p-p38) was inhibited in Z-138, Mino, and JVM-2 cells infected with shMerTK compared to that in cells infected with shControl. **c** Proliferation of Z-138, Mino, and JVM-2 cells infected with shMerTK was significantly suppressed compared to that in the shControl group. **d** MerTK knockdown significantly suppressed invasive abilities of Z-138, Mino, and JVM-2 cells. Invasive abilities were determined by the number of viable cells invading into the lower chamber of the transwell chambers coated with Matrigel inserted in 24-well plates. Images from a representative experiment are shown. Mean values and standard errors (SEs) were derived from at least three independent experiments. *P < 0.05; **P < 0.01; ***P < 0.001; ****P < 0.0001

The efficacy of MerTK knockdown by shRNA in Z-138, Mino, and JVM-2 cells was validated by western blot (Fig. 2a), which showed that MerTK expression in cells infected with shMerTK was scarcely visible compared with that in the parental cells or cells infected with shControl. Previous data reported that AKT, ERK1/2, p38, STAT6, and FAK are downstream signaling proteins of MerTK, and MerTK inhibition via shRNA or inhibitors reduces activation of the downstream signaling proteins [12, 15]. Similarly, western blot indicated that in Z-138, Mino, and JVM-2 cells infected with shMerTK, phosphorylated AKT and p38 was suppressed compared to that in cells infected with shControl (Fig. 2b).

In cell proliferation assays, viable Z-138 cells with MerTK knockdown were suppressed by 51.7% relative to the shControl cells at 96 h (Fig. 2c). Similarly, proliferation of Mino and JVM-2 cells infected with shMerTK was significantly suppressed by 57.8 and 32.9% relative to the shControl cells at 96 h, respectively. To verify the knockdown effects of shMerTK on MCL cells, another lentiviral shRNA targeting MerTK (shMerTK 4) was constructed. Consistent with the previous results mediated by shMerTK, shMerTK4 efficiently inhibited the proliferation and downstream signal pathway (Additional file 3: Figure S1A, B).

For invasive abilities, we adopted transwell chambers coated with Matrigel. Invasive abilities were significantly suppressed in Z-138, Mino, and JVM-2 cells infected with shMerTK respectively by 48.0, 73.8, and 59.7% relative to the shControl cells at 24 h (Fig. 2d). Migration abilities were significantly suppressed in Z-138, Mino, and JVM-2 cells infected with shMerTK respectively by 41.5, 67.6, and 39.7% relative to the shControl cells at 24 h (Additional file 3: Figure S2A, B).

### UNC2250, a novel MerTK inhibitor, reduced activation of downstream signaling and inhibited proliferation, invasion, and migration in MCL cells

**Fig. 3:**
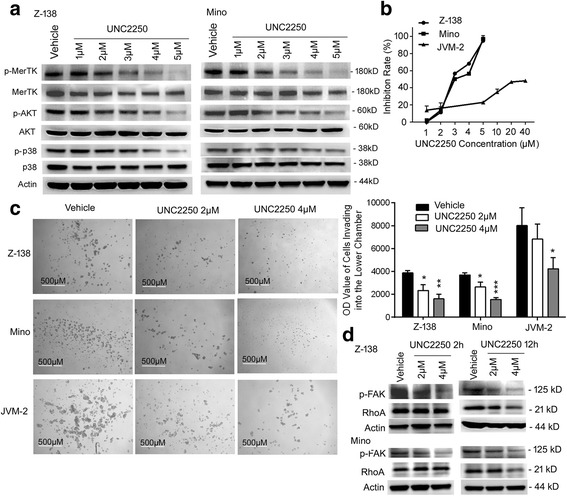
UNC2250 reduced activation of downstream signaling and inhibited proliferation and invasion of MCL cells. **a** UNC2250 induced suppression of MerTK-dependent signaling pathways in a dose-dependent manner. Z-138, Mino, and JVM-2 cells were treated with indicated concentrations of UNC2250 for 60 min; then, whole cell lysates were detected by western blot for phospholated and total MerTK, AKT, and p38. **b** UNC2250 inhibited proliferation of MCL cells in a dose-dependent manner. Cells were cultured in the absence (vehicle) or presence (dosing) of UNC2250 for 72 h. Viable cells were measured by Cell Titer-Glo Luminescent Cell Viability Assay system. Inhibition rates were calculated by (1 − dosing/vehicle) × 100%. **c** UNC2250 suppressed invasion of Z-138, Mino, and JVM-2 cells in a dose-dependent manner. Z-138, Mino, orJVM-2 cells pre-treated with vehicle and 2 or 4 μM UNC2250 for 2 h were seeded into transwell chambers coated with Matrigel inserted in 24-well plates. Invasive abilities were determined by viable cells invading into the lower chamber. Images from a representative experiment are shown. Mean values and SEs were derived from three independent experiments. *P < 0.05; **P < 0.01; ***P < 0.001; ****P < 0.0001. **d** UNC2250 mediated decreases of phospholated FAK and total RhoA in a dose- and time-dependent manner in Z-138 and Mino cells. Cells were treated with indicated concentrations of UNC2250 for 2 or 12 h; then, whole cell lysates were detected by western blot for phospholated FAK and total RhoA. Actin is shown as a loading control

To assess the role of UNC2250 in MerTK-dependent downstream signaling, Z-138 and Mino cells were treated with vehicle or 1, 2, 3, 4, or 5 μM UNC2250 for 1 h. With increasing concentrations of UNC2250 in Z-138 and Mino cells, phosphorylated MerTK was suppressed in a dose-dependent manner and almost vanished at the concentration of 5 μM (Fig. 3a). Moreover, phosphorylated AKT and p38 was also suppressed, consistent with the suppression of phosphorylated MerTK in Z-138 and Mino cells treated with UNC2250 (Fig. 3a).

In MCL cells, UNC2250 inhibited proliferation of Z-138, Mino, and JVM-13 cells, and the IC-50 values respectively were 3.0, 3.1 and 7.7 μM (Fig. 3b, Additional file 3: Figure S3A). For JVM-2 cells, the inhibition of proliferation almost reached a plateau when the concentration of UNC2250 reached 20 μM at 72 h (Fig. 3b). However, we observed JVM-2 cells under a microscope (Nikon, Chiyoda, Tokyo, Japan) and found that cell size increased at 24 h after treatment with 2 μM UNC2250 compared with vehicle- or non-treated cells. We assumed that UNC2250 inhibited cell division and induced polyploidy in JVM-2 cells, which was validated in the following cell cycle analysis. UNC2250 had little effect on the proliferation of Granta519 and JeKo-1 cells (Additional file 3: Figure S3B, C).

For invasion assays, Z-138, Mino, or JVM-2 cells treated with vehicle and 2 or 4 μM UNC2250 for 2 h in FBS-free DMEM were transferred into transwell chambers coated with Matrigel in 24-well plates to be cultured for a further 24 h. UNC2250 (2 and 4 μM) significantly inhibited invasive abilities by 39.5 and 58.6% in Z-138 cells, respectively, and by 28.3 and 58.0% in Mino cells relative to the vehicle-treated cells (Fig. 3c). Only 4 μM UNC2250 induced significant inhibition of invasion, by 47.3%, in JVM-2 cells relative to the vehicle-treated cells. For migration assays, 2 μM UNC2250 inhibited migration abilities in Z-138, Mino, and JVM-2 cells by 42.8, 36.3, and 21.2% relative to the vehicle-treated cells, respectively; 4 μM UNC2250 inhibited migration abilities in Z-138, Mino, and JVM-2 cells by 61.3, 61.3, and 45.2% relative to the vehicle-treated cells, respectively (Additional file 3: Figure S2C, D). To determine the exact mechanism by which MerTK inhibition suppressed invasion and migration of MCL cells, signal proteins associated with invasion and migration were detected by western blot. Consistent with the impaired invasion and migration of MCL cells, phosphorylated FAK was suppressed in Z-138 and Mino cells treated with 2 or 4 μM UNC2250 for 2 h, and total RhoA was suppressed in Z-138 and Mino cells treated with 2 or 4 μM UNC2250 for 12 h (Fig. 3d).

### UNC2250 promoted apoptosis and induced G2/M phase arrest in MCL cells

**Fig. 4:**
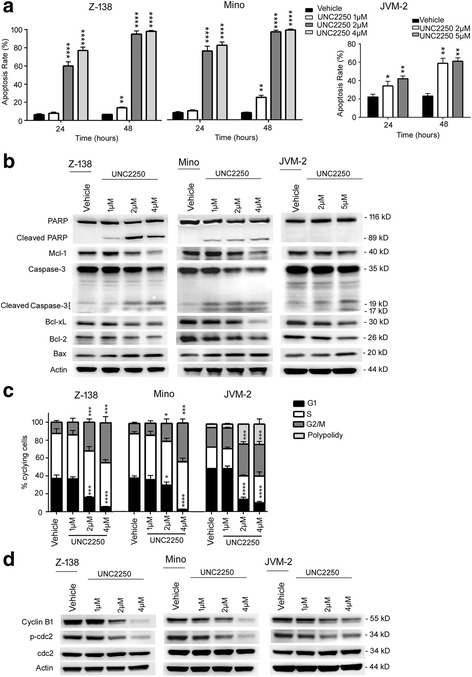
UNC2250 prompted apoptosis and induced G2/M phase arrest in MCL cells. **a** UNC2250 prompted apoptosis in Z-138, Mino, and JVM-2 cells. Cells pre-treated with indicated concentrations of UNC2250 for 24 and 48 h were stained with annexin-V-FITC and propidium iodide (PI). Then, apoptosis assays were performed by flow cytometry. Apoptosis cells were determined by FITC^+^ PI^−^ cells and FITC^+^ PI^+^ cells. **b** UNC2250 mediated alterations in proteins associated with apoptosis in Z-138, Mino, and JVM-2 cells. Cells were treated with indicated concentrations of UNC2250 for 24 h; then, proteins associated with apoptosis were detected by western blot. **c** UNC2250 induced G2/M phase arrest in MCL cells. Z-138, Mino, and JVM-2 cells were treated with indicated concentrations of UNC2250 for 24 h. Then, cell cycle analysis was performed by flow cytometry. **d** UNC2250 mediated alterations in proteins associated with cell cycle in Z-138, Mino, and JVM-2 cells. Cells were treated with indicated concentrations of UNC2250 for 24 h; then, proteins associated with cell cycle were detected by western blot. Mean values and SEs were derived from three independent experiments. *P < 0.05; **P < 0.01; ***P < 0.001; ****P < 0.0001

Apoptosis assays and cell cycle analysis were performed by flow cytometry to assess functional effects of UNC2250-mediated MerTK inhibition in MCL cells. For apoptosis assays, Z-138 and Mino cells were cultured with 1, 2, or 4 μM UNC2250 or vehicle, while JVM-2 cells were cultured with 2 or 5 μM UNC2250 or vehicle, for 12, 24, and 48 h. Flow cytometry analysis revealed that 4 μM UNC2250 induced apoptosis in Z-138 cells by 45.2% and Mino cells by 44.4% at 12 h after treatment (Additional file 3: Figure S2 D). When the time was extended to 24 or 48 h, the apoptosis rates induced by 4 μM UNC2250 increased to 76.9 or 98.0% in Z-138 cells and to 81.5 or 98.1% in Mino cells (Fig. 4a, Additional file 3: Figure S4A, B). For the JVM-2 cells, 2 or 5 μM UNC2250 induced 34.1 or 42.1% apoptosis after 24 h, and 58.8 or 61.1% after 48 h (Fig. 4a, Additional file 3: Figure S4 C). Thus, UNC2250 mediated dose- and time-dependent apoptotic death in Z-138, Mino, and JVM-2 cells. Consistent with the flow cytometry results, proteins associated with apoptosis changed accordingly in Z-138 and Mino cells treated with 1, 2, or 4 μM UNC2250 or vehicle or in JVM-2 cells treated with 2 or 5 μM UNC2250 or vehicle for 24 h. More specifically, Bax, cleaved caspase 3, and PARP protein levels increased with the increasing concentration of UNC2250, whereas Bcl-2, Mcl-1, and Bcl-xL levels decreased (Fig. 4b).

In cell cycle analysis, after being cultured for 24 h, cells in G2/M phase increased from 11.8 to 13.6, 20.5, or 43.8% in Z-138 cells as UNC2250 concentrations increased from 0 (vehicle) to 1, 2, or 4 μM, which correlated with a decrease in cells in G1 phase (Fig. 4c, Additional file 3: Figure S5A). Similarly, UNC2250 also induced G2/M phase arrest in a dose-dependent manner in Mino and JVM-2 cells (Fig. 4c, Additional file 3: Figure S5A). UNC2250 also induced polyploidy in JVM-2 cells. Polyploidy accounted for 22.7 or 23.4% in JVM-2 cells treated with 2 or 4 μM UNC2250, respectively. Expression of cyclin B1 and phosphorylated Cdc2 decreased with increasing concentration of UNC2250 in all three cell lines, which was associated with G2/M phase arrest (Fig. 4d).

### UNC2250 delayed disease progression in MCL-cell-derived xenograft model

**Fig. 5:**
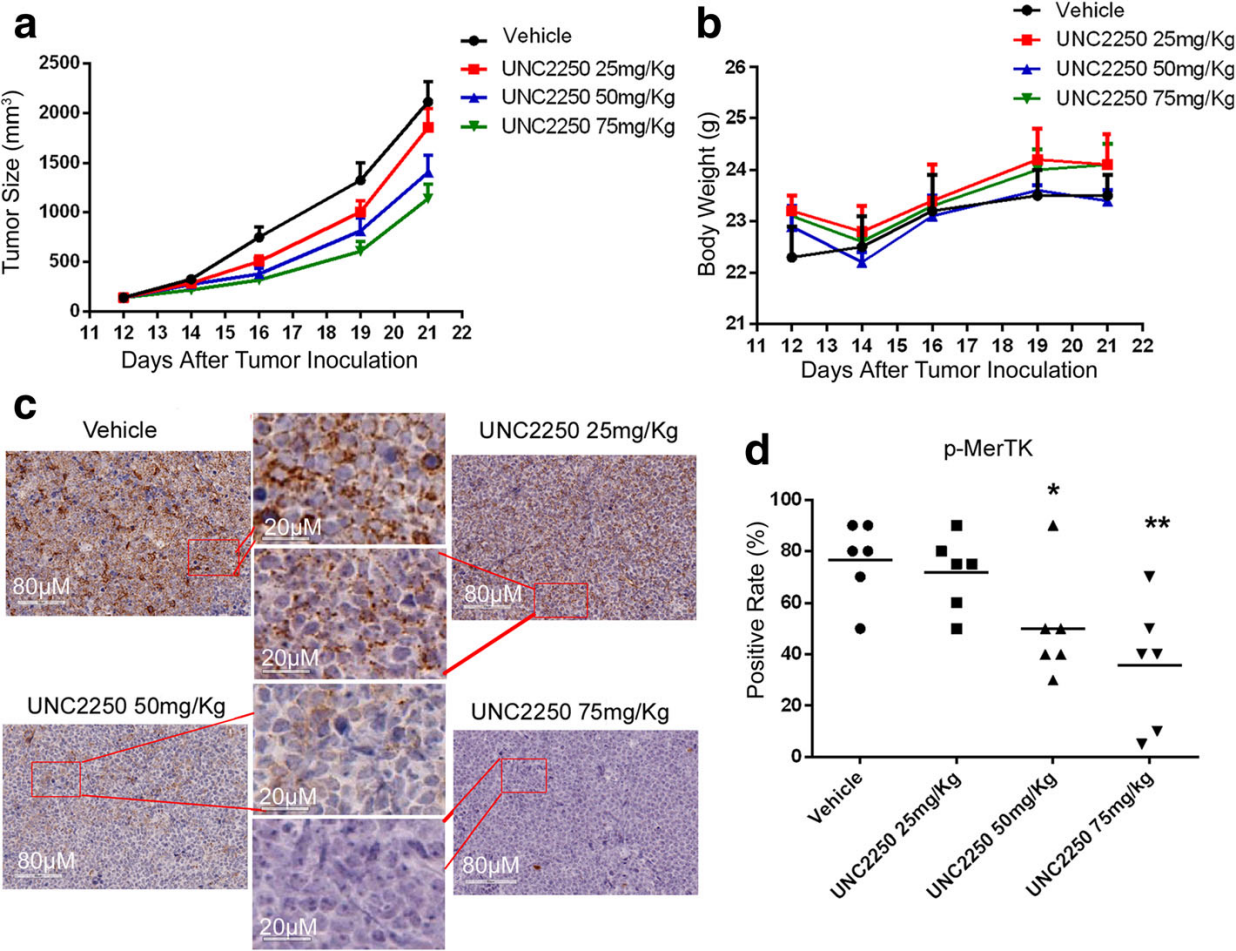
UNC2250 delayed disease progression in MCL-cell-derived xenograft mouse model. UNC2250 (25, 50, or 75 mg/kg) or vehicle was administered once daily by oral gavage to NOD/SCID mice with Z-138-cell-derived xenograft for 10 days. **a** Tumor size curves derived from Z-138 xenograft mouse model. **b** Body weight curves derived from Z-138 xenograft mouse model. **c**, **d** Immunohistochemistry (IHC) assays were performed to detect phosphorylated MerTK (p-MerTK) in tumor tissues from the Z-138 xenograft model treated with vehicle and 25, 50, or 75 mg/kg UNC2250. Representative IHC images from each treatment arm are shown (**c**). Mean values and SEs were derived from each treatment arm (*n* = 6). *P < 0.05; **P < 0.01; ***P < 0.001; ****P < 0.0001

NOD/SCID mice were inoculated with Z-138 cells to establish an MCL-cell-derived xenograft model. UNC2250 (25, 50, and 75 mg/kg) or vehicle was administered once daily by oral gavage. The animal experiment was terminated at 10 days after treatment because average tumor volume of the vehicle group reached 2000 mm^3^. At the end of the experiment, UNC2250 produced significant anti-tumor effects on tumor volume at 50 and 75 mg/kg (*P* = 0.021 and 0.001), and tumor inhibition rate was 35 and 48%, respectively relative to the vehicle-treated group (Fig. 5a). Therefore, UNC2250 mediated a significant dose-dependent reduction in tumor burden relative to the vehicle-treated group. Treatment was well tolerated, and no mice lost weight obviously or died after treatment with any dose of UNC2250 (Fig. 5b). IHC assays revealed that phosphorylated MerTK was significantly reduced in tumor tissues from Z-138-xenograft models treated with 50 mg/kg UNC2250 and 75 mg/kg compared to that in the vehicle-treated group (Fig. 5c, d).

### MerTK inhibition sensitized MCL cells to common chemotherapeutic agents

**Fig. 6:**
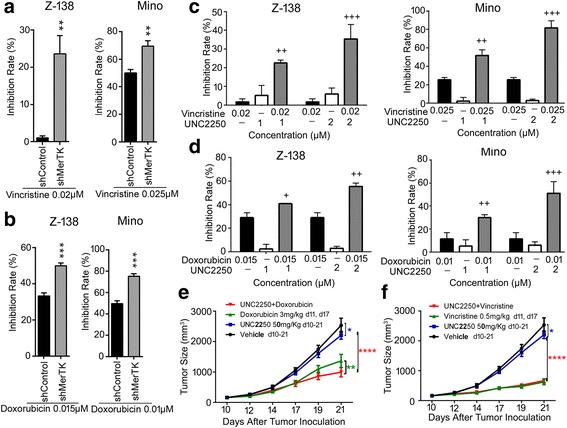
MerTK inhibition sensitized MCL cells to common chemotherapeutic agents. **a**, **b** MerTK knockdown sensitized MCL cells to vincristine and doxorubicin in vitro. Z-138 and Mino cells pre-infected with shControl or shMerTK were treated with indicated concentrations of vincristine (**a**) or doxorubicin (**b**) for 72 h. Inhibition rates were calculated as that in Fig. 3b. Mean values and SEs were derived from three independent experiments. **P < 0.01; ***P < 0.001. **c**, **d** UNC2250 increased the cytotoxic effect of vincristine and doxorubicin in vitro. Z-138 and Mino cells were treated with indicated concentrations of vincristine (**c**) (or doxorubicin (**d**)) alone, UNC2250 alone, or combination of vincristine (or doxorubicin) and UNC2250 for 72 h. Inhibition rates were calculated as that in Fig. 3b. The combination index values (CI) were calculated using CalcuSyn software. + slight synergism (CI 0.85–0.90); ++ moderate synergism (CI 0.7–0.85); +++ synergism (CI 0.3–0.7). **e**, **f** UNC2250 provided additional therapeutic benefit in combination with doxorubicin rather than vincristine in vivo. Tumor size curves derived from Z-138 xenograft mouse model treated with UNC2250 in combination with doxorubicin (**e**) or vincristine (**f**). Mean values and SEs were derived from each treatment arm (*n* = 6). *P < 0.05; **P < 0.01; ****P < 0.0001. (Blue asterisks denote comparison between vehicle and UNC2250 monotherapy, green asterisks denote comparison between indicated treatment and combination therapy, and red asterisks denote comparison between UNC2250 and combination therapy)

Vincristine and doxorubicin are both common agents used in the chemotherapeutic regimens for MCL. To figure out whether MerTK inhibition improved chemosensitivity, Z-138 and Mino cells were treated with vincristine or doxorubicin (Additional file 3: Figure S6). In Z-138 and Mino cells, inhibition rates significantly increased in MerTK knockdown cells treated with vincristine or doxorubicin compared to shControl cells treated with vincristine or doxorubicin (Fig. 6a, b). Inhibition in cells co-treated with UNC2250 and vincristine or doxorubicin significantly increased compared to cells treated with vincristine or doxorubicin alone or cells treated with UNC2250 alone (Fig. 6c, d). UNC2250 and vincristine (or doxorubicin) produced synergistic anti-tumor effects in Z-138 and Mino cells (Additional file 1: Table S3). MerTK inhibition by either MerTK knockdown or treatment with UNC2250 sensitized Z-138 and Mino cells to common chemotherapeutic agents in vitro. To determine whether UNC2250 has a similar effect in vivo, UNC2250 and vincristine or doxorubicin were co-administered to Z-138-xenograft models. The combination treatment produced greater anti-tumor effect than either UNC2250 monotherapy or doxorubicin monotherapy (Fig. 6e), but co-therapy with UNC2250 and vincristine did not show additional therapeutic benefit compared to vincristine monotherapy (Fig. 6f). Thus, MerTK inhibition by treatment with UNC2250 sensitized Z-138 cells to doxorubicin in vivo.

### The expression of microRNA-126 and microRNA-335 in Z-138, Mino, and JVM-2 cells

The reason for ectopic expression of MerTK in MCL remains unclear. Loss of miR-335 and miR-126 was revealed as a possible reason for MerTK overexpression in metastatic breast cancer [27]. Relative microRNA-126 and microRNA-335 expression were detected by real-time PCR in Z-138, Mino, and JVM-2 cells (Additional file 2: Supplementary Methods). Results showed that the three MCL cell lines did not express microRNA-126 and microRNA-335 (Additional file 3: Figure S7 A, B).

### The expression of Gas6 in Z-138, Mino, and JVM-2 cells

Our data demonstrated that MerTK was phosphorylated in MCL cells, to determine the expression status of its corresponding ligand—Gas6; Gas6 mRNA was detected by real-time PCR (Additional file 2: Supplementary Methods); and Gas6 protein was detected by western blot. Results showed that Z-138, Mino, and JVM-2 cells expressed Gas6 at both mRNA and protein level (Additional file 3: Figure S7 C, D), consistent with the expression of MerTK mRNA and MerTK protein.

## Discussion

Most patients with MCL, a clinically aggressive B cell lymphoma, respond to standard first-line therapies but then relapse within a short duration. Despite a wide variety of agents (temsirolimus, bortezomib, ibrutinib, and lenalidomide) being applied in salvage therapies [3–9], outcomes for relapsed and refractory MCL patients are still far from optimistic. Therefore, the search for new therapeutic targets is ongoing to improve prognosis of MCL patients. Here, for the first time, our data showed that ectopic MerTK expression was seen in a large proportion of MCL tumor samples and four of six MCL cell lines. To establish the function of MerTK in MCL, we conducted MerTK inhibition in vitro and in vivo, by either shRNA or treatment with UNC2250. MerTK inhibition suppressed activation of downstream pro-survival pathways, proliferation, invasion, and migration in MCL cells. In addition, UNC2250 promoted apoptosis and induced G2/M phase arrest in MCL cells and delayed disease progression in MCL-cell-derived xenograft models. Importantly, MerTK inhibition promoted chemosensitivity to common therapeutic agents both in vitro and in vivo.

Previous studies proved that MerTK is not expressed in normal B and T lymphocytes, but in neoplastic B or T lymphocytes (such as B-ALL and T-ALL) [15, 17], so MerTK may be a therapeutic target in B or T cell-derived malignant tumors. Here, western blot proved that four of six MCL cell lines expressed MerTK ectopically, whereas normal B cells did not express MerTK. For the first time, we analyzed MerTK expression retrospectively in a large number of MCL patients by IHC assays, which revealed that approximately half of patients showed positive expression of MerTK. Loss of miR-335 and miR-126 was revealed as a possible reason for MerTK overexpression in metastatic breast cancer [27], but our data demonstrated that Z-138, Mino, and JVM-2 cells did not express microRNA-126 and microRNA-335. Further studies are needed to establish the exact mechanism of aberrant MerTK expression in MCL and other malignant tumors. The high expression rate of MerTK in MCL patients provides a specific and applicable population for MerTK targeting therapy. Because first-line therapies affect the prognosis of MCL patients, we only analyzed the relationship between survival and MerTK expression in patients receiving R-CHOP-like regimens. Even though survival analysis indicated that MerTK expression had little effect on OS and PFS of MCL patients who received R-CHOP-like regimens, median OS of the MerTK-positive group (36.5 months) was shorter than the 53.2 months of the MerTK-negative group, which indicated that MCL patients with MerTK expression may experience shorter OS. Studies with larger samples are needed to analyze further the relationship between MerTK expression and prognosis of MCL patients.

Our data showed that Z-138, Mino, and JVM-2 cells expressed Gas6 at both mRNA and protein level. Therefore, MerTK may be activated in MCL cells by its corresponding ligand (Gas6) in an autocrine action [28, 29]. AKT and p38 are commonly activated in MCL tumors [30, 31], and elevated levels of phosphorylated AKT and p38 are associated with shorter survival in MCL [30, 32]. We demonstrated that MerTK inhibition by shRNA or increasing doses of UNC2250 suppressed activation of AKT and p38, thus blocking key nodes of proliferation and pro-survival-related signaling in MCL. Two lentiviral shRNA targeting MerTK (shMerTK and shMerTK 4) were constructed and applied in MerTK knockdown assays, and results of the downstream signaling assays and cell proliferation assays were consistent between the two lentiviral shRNA, which demonstrated that the effects of lentiviral shRNA are indeed due to MerTK knockdown but not due to off-target effects of shRNA. UNC2250 significantly suppressed proliferation of MerTK-positive Z-138, Mino, JVM-2, and JVM-13 cells, but had little effect on MerTK-negative JeKo-1 and Granta519 cells; thus, the effects of UNC2250 on MCL cells are MerTK specific and not due to off-target inhibition. UNC2250 induced different response in suppression of proliferation in JVM-2 cells compared with the other MerTK-positive cell lines; the possible reason is that JVM-2 cell line is a bimodal female cell line immortalized in vitro with Epstein-Barr virus (EBV) with approximately half the cells being pseudodiploid and the other half pseudotetraploid [33].

MerTK is closely related to tumor cell migration and invasion. In melanoma and glioblastoma cells, MerTK inhibition mediated abrogation of migration and invasion by altering signaling through total and phosphorylated FAK, RhoA, and total and phosphorylated myosin light chain 2 (MLC2) [12, 34, 35]. In this study, all MCL cell lines are suspension cells; thus, invasive abilities of MCL cells were determined by the ability of secreting secreted extracellular hydrolytic enzymes to digest Matrigel and pass through the membrane to enter the lower chamber. Consistent with previous reports about MerTK in other malignant tumors, MerTK inhibition by either shRNA or treatment with UNC2250 attenuated invasion and migration in MCL cells, accompanied by decrease in phosphorylated FAK and total RhoA.

MerTK not only modulates activation of downstream signaling, but also regulates gene transcription [15]. In B-ALL, knockdown of MerTK attenuates expression of pro-survival protein kinase C and increases expression of pro-apoptotic proteins (BAX and PUMA) [15]. Here, we demonstrated that MerTK inhibition by treatment with UNC2250 led to increased expression of pro-apoptotic proteins, including Bax, cleaved caspase 3, and PARP, and decreased expression of pro-survival proteins, including Bcl-2, Mcl-1, and Bcl-xL in MCL cells. For JVM-2 cells, we did not detect cleaved PARP when apoptosis was obvious, so apoptosis in JVM-2 cells may not depend on the classical caspase pathway. Furthermore, cell-cycle-associated proteins including cyclin B1 and phosphorylated Cdc-2 also decreased due to MerTK inhibition by treatment with UNC2250, accompanied by G2/M phase arrest in MCL cells.

Although MerTK inhibition by shRNA or treatment with UNC2250 suppressed oncogenic potential of MCL cells in vitro, UNC2250 mediated potent but limited effects on MCL-cell-derived xenograft mouse models. UNC2250 at a dose of 75 mg/kg produced ~ 50% inhibition of tumor growth relative to the vehicle-treated group in MCL-cell-derived xenograft mouse models, indicating that effects of UNC2250 monotherapy were limited. However, we demonstrated here that MerTK inhibition sensitized MCL cells to treatment with vincristine in vitro and doxorubicin both in vitro and in vivo. Thus, combination of MerTK inhibition and common chemotherapeutic agents produced greater effects on MCL cells, suggesting that co-treatment with MerTK inhibition and common chemotherapeutic agents could be a new strategy to treat MCL.

## Conclusions

In summary, our data revealed that MerTK was ectopically expressed in MCL. MerTK inhibition by either shRNA or treatment with UNC2250 apparently suppressed oncogenic potential in MCL cells, delayed disease progression in MCL-cell-derived xenograft models, and prompted chemosensitivity to vincristine and doxorubicin in vitro or in vivo. Therefore, ectopic MerTK may be a novel therapeutic target in MCL, and it is essential to perform pre-clinical or even clinical studies on UNC2250 or new MerTK inhibitors.

## Additional files

Additional file 1: **Table S1.** Baseline characteristics of MCL patients receiving R-CHOP like regimens and their correlations with MerTK. **Table S2.** Information of antibodies applied in immunohistochemistry and western blot assays. **Table S3.** Combination index values of UNC2250 and Vincristine or Doxorubicin in Z-138 and Mino cells. (DOCX 28 kb)

Additional file 2: Supplementary Methods: Confocal immunofluorescence assays; RNA extraction, reverse transcription and Real-Time PCR. (DOCX 2252 kb)

Additional file 3: **Figure S1.** MerTK knockdown mediated by shMerTK 4 suppressed downstream signaling pathways and proliferation in MCL cells. **Figure S2.** MerTK inhibition by either shRNA or treatment with UNC2250 suppressed migration of MCL cells. **Figure S3.** The effects of UNC2250 on proliferation and apoptosis of MCL cells. **Figure S4.** Representative flow cytometry profiles for apoptosis assays in Z-138, Mino and JVM-2 cells. **Figure S5.** Representative flow cytometry profiles for cell cycle analysis in Z-138, Mino and JVM-2 cells. **Figure S6.** Proliferation of Z-138 and Mino cells was inhibited with increasing concentrations of vincristine or doxorubicin. **Figure S7.** Expression of microRNA-126, microRNA-335 and Gas6 in MCL cells. (DOCX 15 kb)

## Abbreviations

AML: Acute myeloid leukemia
ASCT: Autologous hematopoietic stem cell transplantation
B-ALL: Pre B cell acute lymphoblastic leukemia
EBV: Epstein-Barr virus
ERK1/2: Extracellular signal-regulated kinase1/2
FAK: Focal adhesion kinase
FBS: Fetal bovine serum
IHC: Immunohistochemistry
MCL: Mantle cell lymphoma
MerTK: Mer tyrosine kinase
NOD/SCID: Non-obese diabetic/severe combined immunodeficient
OS: Overall survival
PARP: Poly (ADP-ribose) polymerase
PBMCs: Peripheral blood mononuclear cells
PFS: Progression-free survival
PI: Propidiumiodide
R-CHOP: Rituximab-cyclophosphamide, doxorubicin, vincristine, dexamethasone
RTK: Receptor tyrosine kinase
shRNA: Short hairpin RNA
STAT6: Signal transducer and activator of transcription 6
T-ALL: T cell acute lymphoblastic leukemia
