# Supplementary material for: The proto-oncogene Mer tyrosine kinase is a novel therapeutic target in mantle cell lymphoma
Source: J Hematol Oncol. 2018 Mar 20;11:43. doi: 10.1186/s13045-018-0584-6 (PMC5859520; doi:10.1186/s13045-018-0584-6)
Supplement: Supplementary file 1 — Table S1. Baseline characteristics of MCL patients receiving R-CHOP like regimens and their correlations with MerTK. Table S2. Information of antibodies applied in immunohistochemistry and western blot assays. Table S3. Combination index values of UNC2250 and Vincristine or Doxorubicin in Z-138 and Mino cells. (DOCX 28 kb) [file 13045_2018_584_MOESM1_ESM.docx]

Table S1. Baseline characteristics of MCL patients receiving R-CHOP like regimens and their correlations with MerTK.

|  | NO. | MerTK | | | P |
| --- | --- | --- | --- | --- | --- |
|  |  | Negative | | Positive |  |
| Age |  |  |  | |  |
| ≤60 | 27 | 11 | 16 | | 0.34 |
| >60 | 28 | 15 | 13 | |  |
| Sex |  |  |  | |  |
| Male | 41 | 17 | 24 | | 0.21 |
| Female | 14 | 9 | 5 | |  |
| Stage |  |  |  | |  |
| I-II | 5 | 1 | 4 | | 0.36 |
| III-IV | 50 | 25 | 25 | |  |
| B symptoms |  |  |  | |  |
| Negative | 29 | 15 | 14 | | 0.49 |
| Positive | 26 | 11 | 15 | |  |
| LDH |  |  |  | |  |
| Negative | 39 | 17 | 22 | | 0.39 |
| Positive | 16 | 9 | 7 | |  |
| β2-MG |  |  |  | |  |
| Negative | 24 | 13 | 11 | | 0.35 |
| Positive | 29 | 12 | 17 | |  |
| Ki67 |  |  |  | |  |
| ≤25% | 23 | 12 | 11 | | 0.54 |
| >25% | 32 | 14 | 18 | |  |
| IPI |  |  |  | |  |
| 0-2 | 35 | 16 | 19 | | 0.76 |
| 3-5 | 20 | 10 | 10 | |  |
| ESR |  |  |  | |  |
| Negative | 20 | 9 | 11 | | 0.47 |
| Positive | 27 | 15 | 12 | |  |
| HBV |  |  |  | |  |
| Negative | 30 | 17 | 13 | | 0.13 |
| Positive | 25 | 9 | 16 | |  |
| Response* |  |  |  | |  |
| CR | 18 | 9 | 9 | | 0.78 |
| PR+SD+PD | 37 | 17 | 20 | |  |
| OR | 31 | 12 | 19 | | 0.15 |
| SD+PD | 24 | 14 | 10 | |  |

LDH, lactate dehydrogenase; β2-MG, β2-microglobulin; ESR, erythrocyte sedimentation rate; IPI, International Prognostic Index; HBV, hepatitis-B virus;

* Clinical response to R-CHOP therapy: CR, complete response; PR, partial response; OR, overall response; SD, stable disease; PD, progressive disease.

Table S2. Information of antibodies applied in immunohistochemistry and western blot assays.

| Name(anti-) | Cat NO. | Concentration | Application |  |
| --- | --- | --- | --- | --- |
| MerTK | Ab52968 | 1:2000  1:500 | Western blot  IHC | Abcam |
| phospho -Y749 + Y753 + Y754 -MerTK | Ab14921 | 1:1000 | Western blot | Abcam |
| Phospho-Y681 + Y749-MerTK | Ab192649 | 1:100 | IHC | Abcam |
| AKT | #9272 | 1:3000 | Western blot | CST |
| Phospho-Ser473-AKT | #9271 | 1:3000 | Western blot | CST |
| P38 MAPK | #8690 | 1:3000 | Western blot | CST |
| Phospho-Thr180/Tyr182-P38 MAPK | #4511 | 1:3000 | Western blot | CST |
| Actin | A5441 | 1:5000 | Western blot | Sigma-Aldrich |
| PARP | 556362 | 1:3000 | Western blot | BD Bioscience |
| Mcl-1 | #5453 | 1:3000 | Western blot | CST |
| Caspase 3 | #9662 | 1:3000 | Western blot | CST |
| Bcl-2 | #4223 | 1:3000 | Western blot | CST |
| Bcl-xL | #2796s | 1:3000 | Western blot | CST |
| Bax | #5023 | 1:3000 | Western blot | CST |
| Cyclin B1 | #12231 | 1:3000 | Western blot | CST |
| Cdc2 | #9116 | 1:3000 | Western blot | CST |
| Phospho- Tyr15-Cdc2 | #9111 | 1:3000 | Western blot | CST |
| Phospho-Tyr397-FAK | #8556 | 1:3000 | Western blot | CST |
| RhoA | #2117 | 1:3000 | Western blot | CST |
| Gas6 | 13795-1-AP | 1:1000 | Western blot | Proteintech |
| goat-anti-mouse IgG HRP | 1706516 | 1:5000 | Western blot | BioRad |
| goat-anti-rabbit IgG-HRP | 1706515 | 1:5000 | Western blot | BioRad |

Abcam, Cambridge, UK; CST, Cell Signaling Technoligy, Danvers, MA, USA; Sigma-Aldrich, Darmstadt, Germany; BioRad, Hercules, CA, USA. Proteintech Group, Inc, Rosemont, IL, USA

Table S3. Combination index values of UNC2250 and Vincristine or Doxorubicin in Z-138 and Mino cells.

|  | Z-138 | |  | Mino | | |
| --- | --- | --- | --- | --- | --- | --- |
|  | UNC2250(uM) | CI |  | | UNC2250(uM) | CI |
| Vin 0.02uM | 1 | 0.76 | Vin 0.025uM | | 1 | 0.75 |
|  | 2 | 0.68 |  |  | 2 | 0.53 |
| Dox 0.015uM | 1 | 0.88 | Dox 0.01uM | | 1 | 0.72 |
|  | 2 | 0.71 |  |  | 2 | 0.55 |

Combination index (CI) values of UNC2250 and Vincristine or Doxorubicin in Z-138 and Mino cells were calculated using CalcuSyn software. Vin, Vincristine; Dox, Doxorubicin; CI, combination index, CI < 1, = 1, and >1 indicate synergism, additive effect and antagonism, respectively. CI: 0.85-0.90 denotes slight synergism; CI: 0.7-0.85 denotes moderate synergism; CI: 0.3-0.7 denotes synergism.
