# Supplementary material for: The proto-oncogene Mer tyrosine kinase is a novel therapeutic target in mantle cell lymphoma
Source: J Hematol Oncol. 2018 Mar 20;11:43. doi: 10.1186/s13045-018-0584-6 (PMC5859520; doi:10.1186/s13045-018-0584-6)
Supplement: Supplementary file 2 — Supplementary Methods: Confocal immunofluorescence assays; RNA extraction, reverse transcription and Real-Time PCR. (DOCX 2252 kb) [file 13045_2018_584_MOESM2_ESM.docx]

**Supplementary Methods**

***Confocal immunofluorescence assays***

Cells harvested in 1.5 ml EP tube were incubated with pre-cooled methanol for 10 minutes at room temperature (RT) and washed with PBS. Then cells were permeabilized with 0.5% Trition X-100 for 20 minutes at RT and washed with PBS. Afterwards, cells were incubated successively with primary antibodies (MerTK, # 4319, Cell Signaling Technology, Danvers, MA, USA) for 1 hour and Alexa Fluor 594 - conjugated secondary antibodies (ZF-0516, ZSGB-Bio, Beijing, China) for 30 minutes at RT (both prepared in 5% bovine serum albumin (BSA) at 1:100), and then stained with 0.5ug/ml DAPI (Beyotime Biotechnology, Shanghai, China) for 5 minutes. For immunofluorescence analysis, cells were re-suspended with PBS and pipetted onto coverslips.

***RNA extraction,*** ***reverse transcription and Real-Time PCR***

Extraction of total RNA was conducted using Trizol reagent (Life Technologies, Carlsbad, CA, USA) according to the manufacturer’s instructions. For microRNA quantitation only, total RNA was capped Poly (A) tailing using E. coli Poly (A) Polymerase (New England Biolabs Inc., MA, USA). TransScript First-Strand cDNA Synthesis SuperMix (TransGen Biotech, Beijing, China) was used for reverse transcription, and the reverse primer for microRNA reverse transcription is GCGAGCACAGAATTAATACGACTCACTATAGGTTTTTTTTTTTTVN. Real-Time PCR was performed using Go Taq qPCR Master Mix (Promega Corporation, Madison, USA). GAPDH was used as a reference gene for mRNA quantitation, and U6 was used as a reference gene for microRNA quantitation. The fold change in mRNA was calculated by the 2^-∆∆Ct^ method.

The primers for Real-Time PCR were as follows:

MerTK Forward Primer: AAGCAGAGGAGGATGGGTC, Reverse Primer: GAACATTCCGCTCCGACA;

Gas6 Forward Primer: CAATCTCTGTTGAGGAGCTGG, Reverse Primer: GACCACGTGCTCTTGGCCGTC;

GAPDH Forward Primer: GCACCGTCAAGGCTGAGAAC, Reverse Primer: TGGTGAAGACGCCAGTGGA;

Mature microRNA-126 Primer: TCGTACCGTGAGTAATAATGCG;

Mature microRNA-335 Primer: TCAAGAGCAATAACGAAAAATGT;

Reverse Primer for microRNA quantitation: GCGAGCACAGAATTAATACGAC;

U6 Forward Primer: CGCTTCGGCAGCACATATAC, Reverse Primer: TTCACGAATTTGCGTGTCAT.
