# Supplementary material for: The proto-oncogene Mer tyrosine kinase is a novel therapeutic target in mantle cell lymphoma
Source: J Hematol Oncol. 2018 Mar 20;11:43. doi: 10.1186/s13045-018-0584-6 (PMC5859520; doi:10.1186/s13045-018-0584-6)
Supplement: Supplementary file 3 — Figure S1. MerTK knockdown mediated by shMerTK 4 suppressed downstream signaling pathways and proliferation in MCL cells. Figure S2. MerTK inhibition by either shRNA or treatment with UNC2250 suppressed migration of MCL cells. Figure S3. The effects of UNC2250 on proliferation and apoptosis of MCL cells. Figure S4. Representative flow cytometry profiles for apoptosis assays in Z-138, Mino and JVM-2 cells. Figure S5. Representative flow cytometry profiles for cell cycle analysis in Z-138, Mino and JVM-2 cells. Figure S6. Proliferation of Z-138 and Mino cells was inhibited with increasing concentrations of vincristine or doxorubicin. Figure S7. Expression of microRNA-126, microRNA-335 and Gas6 in MCL cells. (DOCX 15 kb) [file 13045_2018_584_MOESM3_ESM.docx]

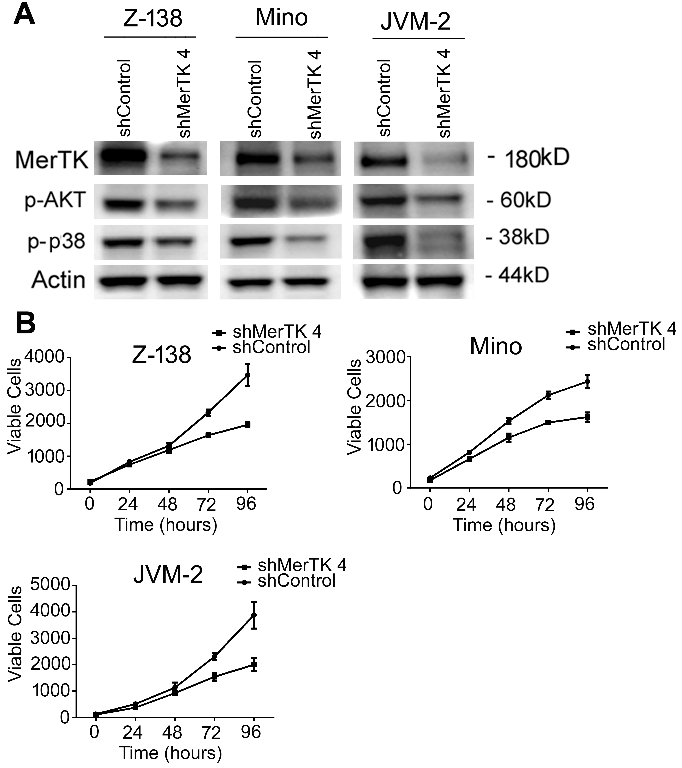


**Figure S1.** MerTK knockdown mediated by shMerTK 4 suppressed downstream signaling pathways and proliferation in MCL cells. A. MerTK knockdown by shMerTK 4 suppressed downstream signaling in Z-138, Mino and JVM-2 cells. Cells were infected with shControl or shMerTK 4, then whole cell lysates were detected by western blot for MerTK, phospholated AKT (p-AKT) and p38 (p-p38). Actin is shown as a loading control. B. Proliferation of Z-138, Mino and JVM-2 cells infected with shMerTK 4 was significantly suppressed compared to that in the shControl group.





**Figure S2.** MerTK inhibition by either shRNA or treatment with UNC2250 suppressed migration of MCL cells. Migration abilities was determined by the number of viable cells invading into the lower chamber. A and B. MerTK knockdown suppressed migration of MCL cells. Z-138, Mino or JVM-2 cells pre-infected with shControl or shMerTK were seeded into transwell chambers without Matrigel in 24-well plates to be cultured for a further 24 hours. Images from a representative experiment are shown (A). Mean values and SEs were derived from three independent experiments (B). *P<0.05; **P<0.01. C and D. UNC2250 suppressed migration of MCL cells in a dose-dependent manner. Z-138, Mino or JVM-2 cells pre-treated with vehicle, 2 or 4 μM UNC2250 for 2 hours were transferred into transwell chambers without Matrigel in 24-well plates to be cultured for a further 24 hours. Images from a representative experiment are shown (C). Mean values and SEs were derived from three independent experiments (D). *P<0.05; **P<0.01.


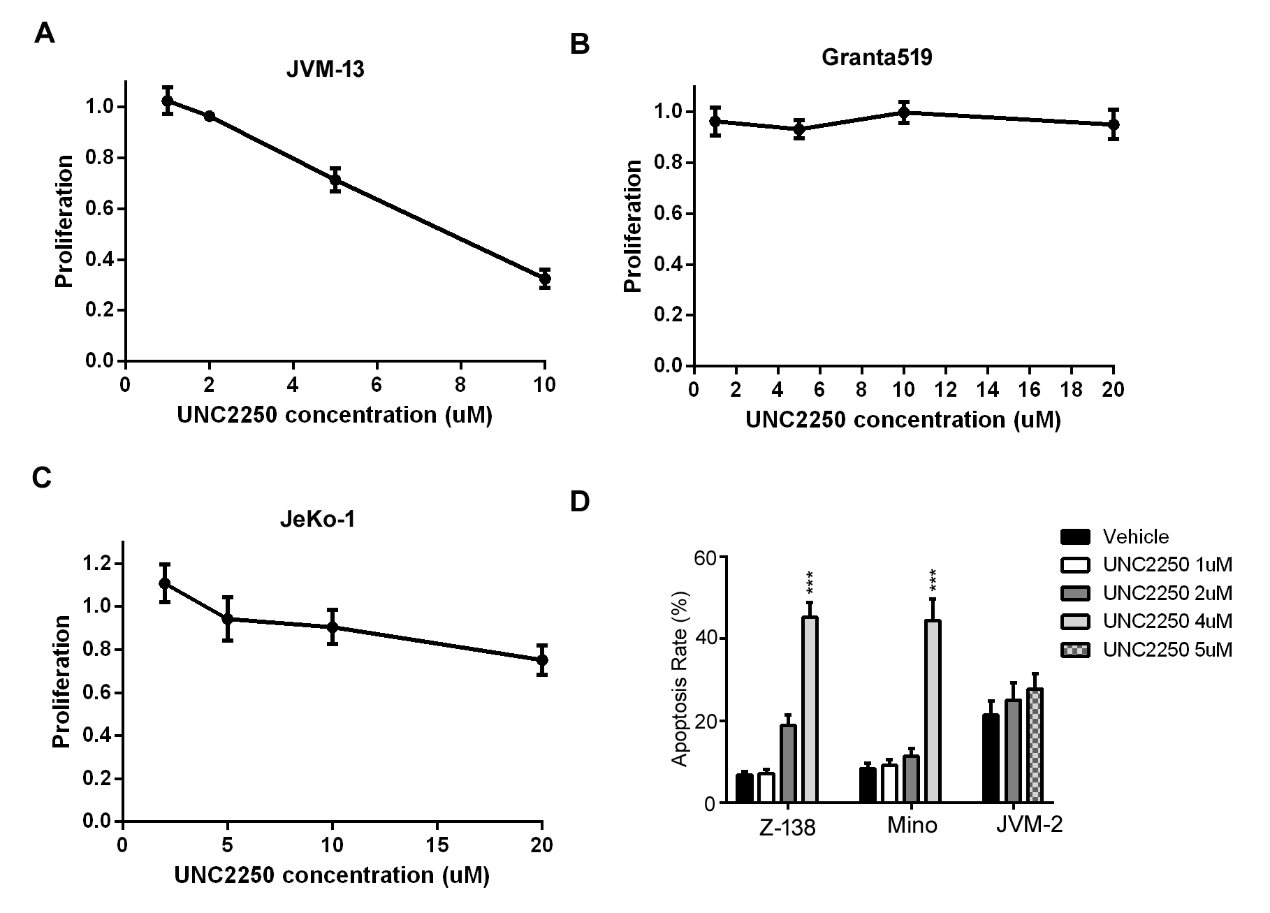


**Figure S3.** The effects of UNC2250 on proliferation and apoptosis of MCL cells. A, B and C. The effects of UNC2250 on proliferation of MCL cells. JVM-13 (A), Granta519 (B) and JeKo-1 (C) were treated with indicated concentrations of UNC2250. Viable cells were measured by Cell Titer-Glo Luminescent Cell Viability Assay system. Proliferation rates were calculated by (Dosing/Vehicle) × 100%. D. UNC2250 prompted apoptosis of MCL cells. Z-138, Mino and JVM-2 cells were cultured with indicated concentrations UNC2250 for 12 hours. Cells were stained with Annexin-V-FITC and propidium iodide (PI). Then apoptosis assays were performed by ﬂow cytometry. Apoptosis cells were determined by FITC^+^ PI^-^ cells and FITC^+^ PI^+^ cells. Mean values and SEs were derived from three independent experiments. ***P<0.001.


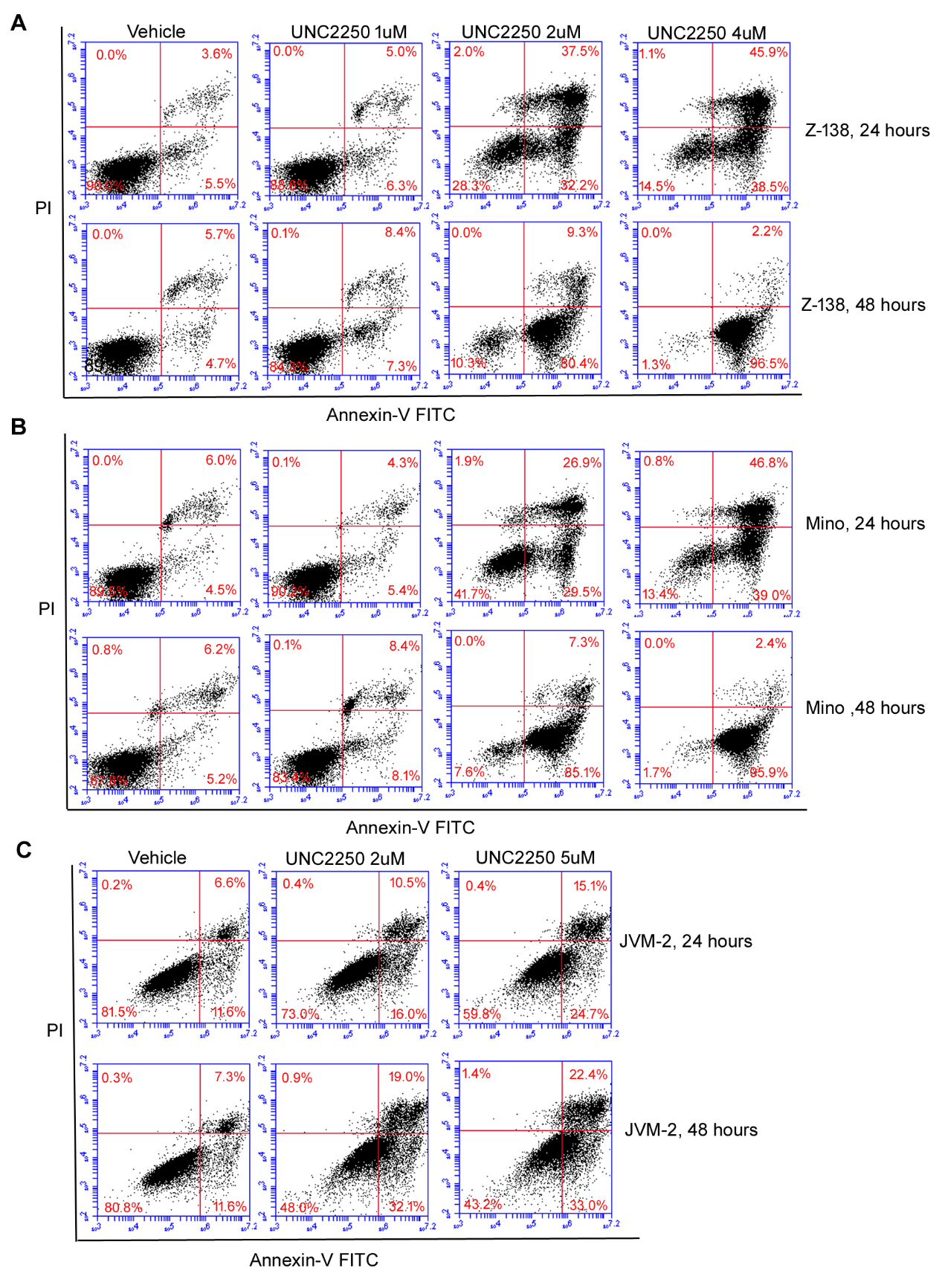


**Figure S4.** Representative flow cytometry profiles for apoptosis assays in Z-138, Mino and JVM-2 cells.


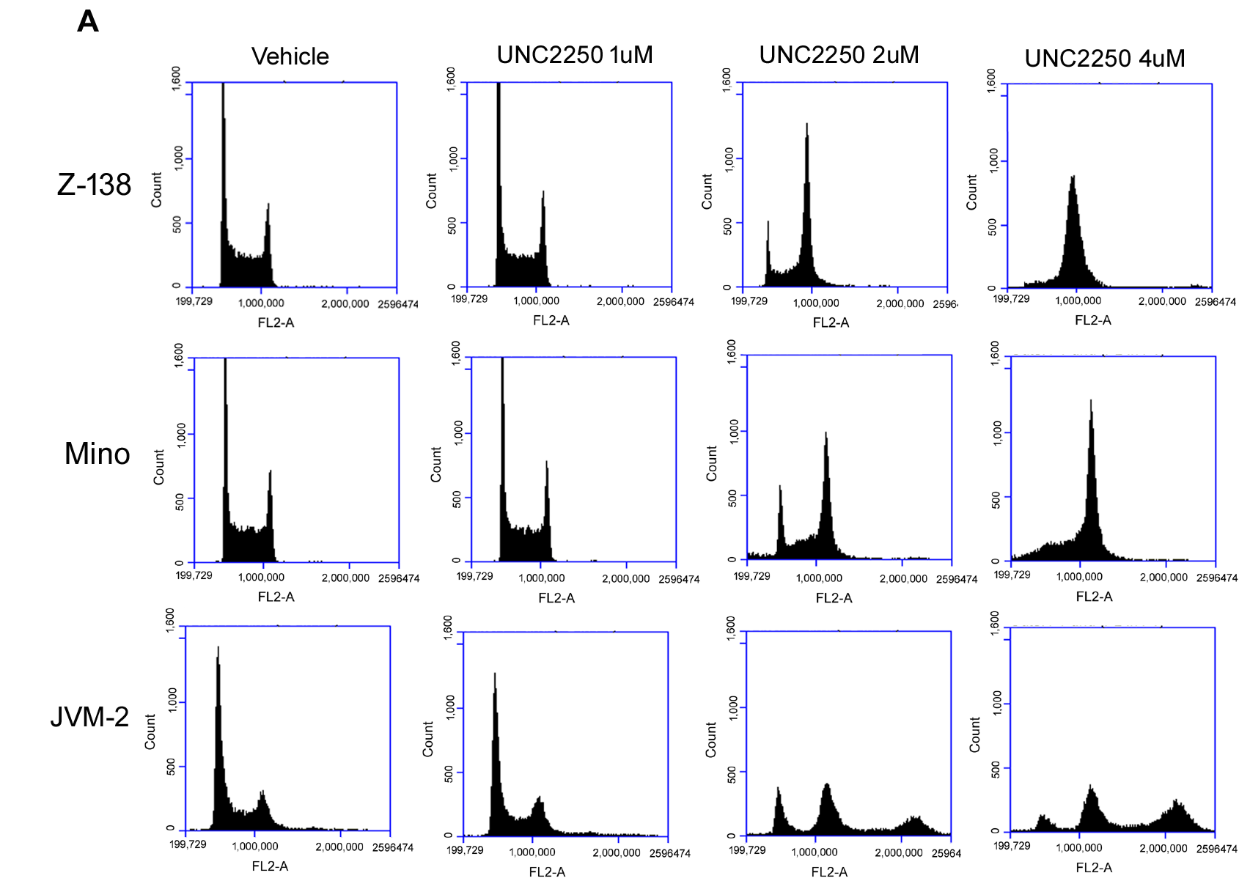


**Figure S5.** Representative flow cytometry profiles for cell cycle anslysis in Z-138, Mino and JVM-2 cells.

**
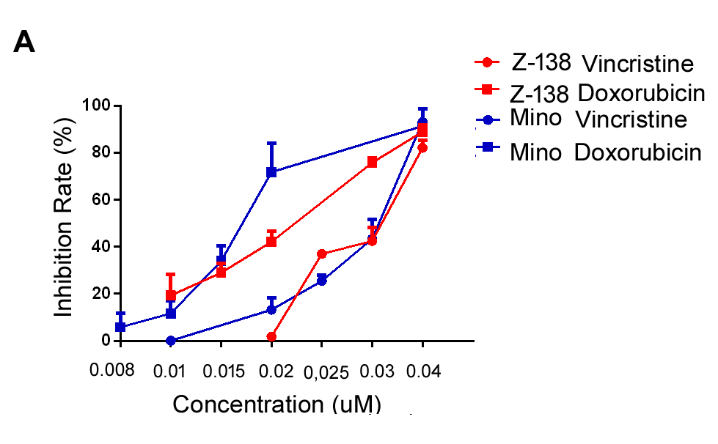
**

**Figure S6.** Proliferation of Z-138 and Mino cells was inhibited with increasing concentrations of vincristine or doxorubicin. Z-138 and Mino cells were treated with indicated concentrations of vincristine or doxorubicin (Dosing) for 72 hours. Viable cells were measured by Cell Titer-Glo Luminescent Cell Viability Assay system. Inhibition rates were calculated by (1-Dosing/Vehicle) × 100%.


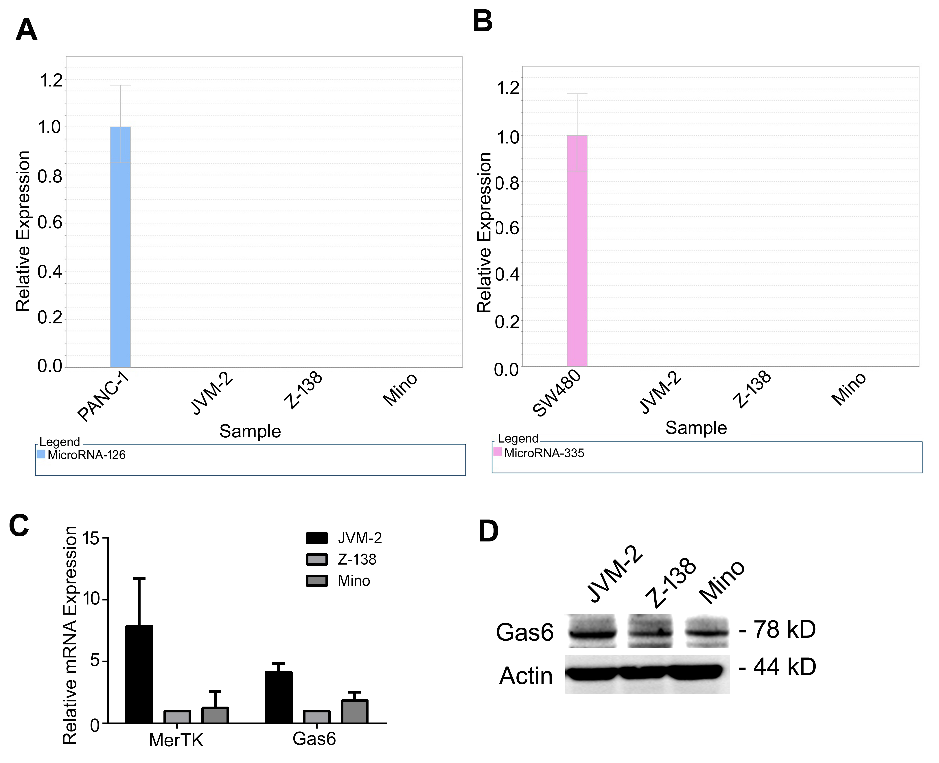


**Figure S7.** Expression of microRNA-126, microRNA-335 and Gas6 in MCL cells. A and B. MicroRNA-126 and microRNA-335 were not expressed in Z-138, Mino and JVM-2 cells. Relative microRNA-126 (A) or microRNA-335 (B) expression were detected by real-time PCR, and U6 was used as a reference gene. PANC-1 cell line was used as positive control for microRNA-126, and SW480 cell line was used as positive control for microRNA-335. C and D. Z-138, Mino and JVM-2 cells expressed Gas6 at both mRNA and protein level. C. Relative MerTK mRNA and Gas6 mRNA expression were detected by real-time PCR, and GAPDH was used as a reference gene. D. Expression of Gas6 protein in Z-138, Mino and JVM-2 cells was detected by western blot. Mean values and SEs were derived from three independent experiments.
